# Spatially Resolved Cross-Linking Characterization by Imaging Low-Coherence Interferometry †

**DOI:** 10.3390/s19051152

**Published:** 2019-03-07

**Authors:** Christopher Taudt, Bryan Nelsen, Elisabeth Rossegger, Sandra Schlögl, Edmund Koch, Peter Hartmann

**Affiliations:** 1 Faculty of Physical Engineering/Computer Sciences, University of Applied Sciences Zwickau, D-08056 Zwickau, Germany; bryan.nelsen@fh-zwickau.de (B.N.); peter.hartmann@fh-zwickau.de (P.H.); 2 Fraunhofer Application Center for Optical Metrology and Surface Technologies, D-08056 Zwickau, Germany; 3 Faculty of Electrical and Computer Engineering, Technical University Dresden, D-01307 Dresden, Germany; edmund.koch@tu-dresden.de; 4 Polymer Competence Center Leoben, AT-8700 Leoben, Austria; elisabeth.rossegger@pccl.at (E.R.); Sandra.Schloegl@pccl.at (S.S.); 5 Faculty of Medicine Carl Gustav Carus, Technical University Dresden, D-01307 Dresden, Germany

**Keywords:** interferometry, cross-linking characterization, white-light interferometry, dispersion-enhanced low-coherence interferometry, photoresist, semiconductor manufacturing

## Abstract

A method to characterize cross-linking differences in polymers such as waveguide polymers has been developed. The method is based on the scan-free information acquisition utilizing a low-coherence interferometer in conjunction with an imaging spectrometer. By the introduction of a novel analyzing algorithm, the recorded spectral-phase data was interpreted as wavelength-dependent optical thickness which is matchable with the refractive index and therefore with the degree of cross-linking. In the course of this work, the method was described in its hardware and algorithmic implementation as well as in its accuracy. Comparative measurements and error estimations showed an accuracy in the range of 10^−6^ in terms of the refractive index. Finally, photo-lithographically produced samples with laterally defined cross-linking differences have been characterized. It could be shown, that differences in the optical thickness of ±1.5 μm are distinguishable.

## 1. Introduction

Polymer-based optical waveguides are usually processed by patterning (photoresist-based or direct lithography), soft lithography or printing techniques [1,2] in order to achieve defined cross-linking and refractive index differences. For their fabrication, polymers have to fulfill several requirements such as optical transparency and chemical as well as thermal stability [3,4]. Advancing from conventional thermoplastics such as polymethyl methacrylate, polystyrene, polycarbonate and polyurethane, research has been geared towards the development of new polymers, which exhibit lower absorption losses and higher stability [1]. Promising classes of polymers are halogenated polyacrylates [5], fluorinated polyimides [6] or polysiloxanes [7].

State-of-the-art cross-linking characterization technologies often require slow, lab-based approaches which are not able to deliver spatial information on a specific sample [8,9]. A very common method to determine the degree of cross-linking is Soxhlet-type extraction [10]. Oreski et al. found that the time for the extraction is at least 18 h while additional drying takes another 24 h [11]. Furthermore Hirschl et al. found, that the extraction time and other process parameters can have a huge influence on the repeatability of the measured degree of cross-linking, especially in weakly cross-linked samples. They determined that the repeatability ranges from 2–4% [12]. The method doesn’t enable spatially resolved measurements and samples are tested destructively.

Another established method is differential scanning calorimetry (DSC) [13,14]. A typical measurement cycle in the so called *dual-run mode* takes 2 × 45 min during which a defined heating profile is applied [11]. Hirschl et al. [8] showed in a comparative study, that different approaches for referencing the measurements to other methods might apply and also that errors in the repeatability can be 10% and larger; Especially weakly cross-linked samples require slower heating profiles, hence longer measurement times, and inherently larger errors [15].

Non-destructive measurements can be obtained by using optical metrology such as Raman spectroscopy [16], or luminescence spectroscopy [17]. Recent works have shown that these technologies are able to characterize cross-linking of coatings on solar cells. In a comparative study, Hirschl and co-workers [18] have demonstrated that Raman spectroscopy gives comparable results to classical methods like Soxhlet-extraction. Although, it has to be noted that the measured errors of the degree of cross-linking were up to 15%, especially for samples with weak cross-linking. Furthermore, acquisition times for Raman spectra depend very much on the signal-to-noise ratio of relevant spectral intensity peaks and hence require a large amount of averaging. Recent studies report acquisition times for single-point measurements between 50–100 s [18,19]. Peike et al. [16] point out, that Raman analysis is very material specific and can be complex with different peaks overlaying each other. Additionally, they found that the signal-to-noise ratio decreases with peaks at higher wavelengths as $I_{peak}∼1/\lambda^{4}$. This can be critical for weakly cross-linked material or materials with a low number of reactional groups. A recent work by Schlothauer et al. has qualified luminescence spectroscopy as a tool for cross-linking characterization with an accuracy of 4–6% [17]. However, the method requires a large amount of averaged spectra in a point-by-point scanning fashion. Acquisition times for a 16 × 16 cm^2^ were about 80 min.

From the analysis of reaction kinectics of polymers, it is known that the density of a material as well as its refractive index changes during cross-linking [20]. This relationship can be described by the Lorentz-Lorenz equation [21]. In particular, applications such as polymeric waveguides or direct laser writing on wafers make use of this effect to generate functional properties with refractive index changes of about $10^{-2}$ [22]. Žukauskas et al. applied this effect to generate gradient-index lens elements with a size of 50 × 50 × 10 μm^3^ [19].

In order to characterize these functional properties alongside with the degree of cross-linking and their spatial distribution, new metrology approaches are necessary. Classical optical-coherence tomography has been used to examine structural defects such as bubbles or phase separation during cross-linking by scanning a sample with in a few seconds [23]. Other interferometric techniques such as spectrally-resolved white-light interferometry, frequency domain interferometry or digital holographic interferometry have been utilized to measure refractive indices with accuracies in the range of $10^{-5}$–$10^{-6}$[24,25] as well as mechanical deformations on the nm-scale in material and biomedical engineering [26,27,28]. A method which also allows measurements with spatial resolution (about 17 μm) was published by Guerrero et al. [29]. It is based on a phase estimation of intensity extrema and shows a theoretical refractive index resolution of $10^{-8}$. Shortcomings of the method are the restriction to measurements of the differential refractive index as well as its dependence on intensity measurements which are influenced by noise.

This work presents a novel approach to overcome the shortcomings of traditional characterization approaches regarding spatial resolution and speed. For this purpose, a low-coherence interferometer with an imaging spectrometer is adapted from surface profilometry [30], for spatially resolved cross-linking characterization without the need for mechanical scanning. Furthermore, a new analysis algorithm is presented to calculate refractive index profiles without any a priori knowledge of the underlying model. The refractive index profiles are calculated directly from measured data while simultaneously the surface profile of the sample can be extracted.

## 2. Methodology

### 2.1. Experimental Approach

**Figure 1:**
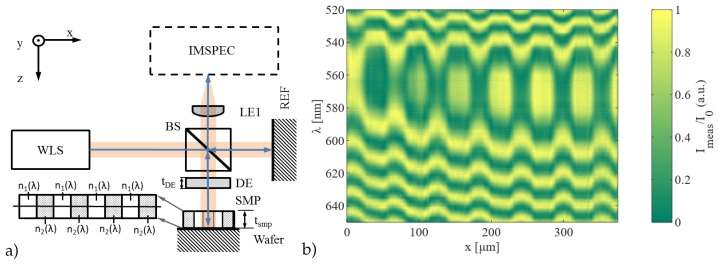
(**a**) Schematic of the measurement setup with WLS—white-light source, BS—beam splitter, REF—fixed reference mirrors, DE—additional dispersive element with thickness $t_{DE}$ and spatially uniform refractive index $n_{DE}$, SMP—sample on a wafer with the thickness $t_{smp}$ and cross section with spatially varying refractive indices $n_{1}\left(\lambda \right)$ and $n_{2}\left(\lambda \right)$, LE1—lens to image the sample on the spectrometer, IMSPEC—imaging spectrometer, where beam paths are marked with blue arrows. (**b**) Example spatial data with the refractive index encoded in the modulation of the measured spectral intensity $I_{meas}$ with respect to the spectral intensity of the light source $I_{0}$ (*y*- and *z*-axis) of a polymer sample with lines and spaces of differently cross-linked sections having a pitch width of 50 μm along a line in the x-dimension.

In order to characterize the degree of cross-linking of a polymeric sample, the sample itself is integrated into one mirror of a Michelson interferometer, Figure 1a.

Specifically, a sample of negative tone photoresist was spin-cast onto a silicon wafer ($t_{smp}=$ 750 μm) and exposed to light using a rectangular patterned mask (pitches of 50 and 100 μm) which produced areas with defined refractive index differences. This sample acts as one mirror in the interferometer setup. During measurement, the light of a white light source (EQ-99X, Energetiq Technology, Inc., Woburn, MA, USA) was split in a 50:50 ratio by a cube beam splitter. In both arms, light traveled the same optical path while the sample under test was placed in one of the arms. After reflection from the wafer and the reference mirror, the recombined signal was imaged onto the slit of an imaging spectrometer by an achromatic lens. The imaging spectrometer was wavelength-calibrated using a gas-discharge lamp and recorded the spectral intensity of the recombined signal for every point on a line of interest, Figure 1b.

By transmitting through the sample volume, the signal was affected by the material dispersion which depends on the wavelength-dependent refractive index n(x,λ) and the thickness $t_{smp}\left(x\right)$. For this reason, the optical path of the corresponding interferometer arm displayed a slight variation in the optical path length for every wavelength. In essence, the signal shows an interference pattern in which the phase inherits a distinct minimum at wavelengths where the optical path difference equals zero in relation to the reference arm, which is called equalization wavelength, $\lambda_{eq}$. This effect can be described with

(1) $$\begin{array}{c} I_{meas}\left(x,\lambda \right)=I_{0}\left(\lambda \right)\;\cdot \;\left(1+\cos\varphi (x,\lambda )\right) \end{array}$$

(2) $$\begin{array}{c} \mathrm{with}\varphi =2\pi \frac{\left[n(x,\lambda )-1\right]t_{smp}-\delta }{\lambda }, \end{array}$$

where $I_{meas}$ describes the measured spectral intensity profile which includes $I_{0}\left(\lambda \right)$, the spectral profile of the light source and φ the phase. In this simple case, it is assumed that the thickness of the sample $t_{smp}$ is uniform and therefore independent of *x*. Furthermore, the optical path difference δ is considered to be equal along the sample with the setup aligned properly in case of the sample surface being flat. As cross-linking of polymers usually comes with shrinkage of the material, $t_{smp}$ should be considered as $t_{smp}\left(x\right)$ accounting for the surface profile of the sample. The described setup enabled the measurement of the surface height profile simultaneously with the determination of the refractive index profile of a sample by combining a low-coherence interferometry approach for profilometry as described in [30] with the analysis algorithm described below. For this purpose, an additional dispersive element (N-BK7, $t_{DE}=$ 4 mm) was introduced into the setup. This additional dispersion is used to tune the measurement range for the surface height profile to Δz= 127 μm [30]. Furthermore, it decreases the spectral width of the signal within one phase jump around $\lambda_{eq}$ which determines the range in which the refractive index can be calculated. The chosen element was the best compromise to enable both measurements. Accordingly, the phase includes an additional, but known term for this material which equals $\left[\left(n_{DE}\left(\lambda \right)-1\right)t_{DE}\right]$.

### 2.2. Data Analysis

In order to estimate the degree of cross-linking, the analysis of the refractive index can be utilized [31]. By rewriting Equation (1), the phase-term containing the refractive index can be extracted,

(3) $$\cos^{-1}\left[\frac{I_{meas}\left(x,\lambda \right)}{I_{0}\left(\lambda \right)}-1\right]=\varphi =2\pi \frac{\left[\left(n_{smp}\left(x,\lambda \right)-1\right)t_{smp}\right]+\left[\left(n_{DE}\left(\lambda \right)-1\right)t_{DE}\right]-\delta }{\lambda }.$$

**Figure 2:**
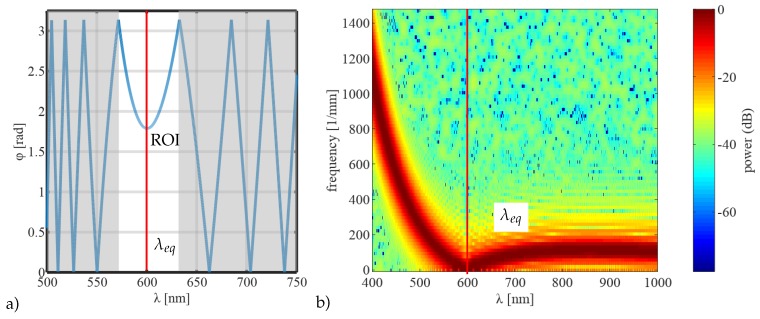
(**a**) Simulated phase data with ambiguities due to the $\cos^{-1}$-operation according to Equation (3) with marked equalization wavelength $\lambda_{eq}$ and region of interest ROI (marked as white band) for the extraction of a local phase and (**b**) power spectrum as a result from Short-time Fourier Transform (STFT) where the phase minimum is determined and used to define the ROI.

Inherent to this approach is the ambiguity of the resulting values as φ is not limited to the range of 0−π. Other works have shown methods to perform the correct quadrant selection in order to resolve this ambiguity [32]. In contrast, we propose a method to avoid quadrant selection by performing a local signal analysis in the spectral range close to $\lambda_{eq}$, Figure 2a.

In the first stage, this approach determines the phase minimum and defines a ROI around the minimum. For this purpose, the raw measured data is analysed using a STFT where a Fast Fourier Transform (FFT) is performed in one small window of the complete data set which is then slid over the signal successively along the wavelength dimension [33]. This approach accounts for the non-uniform frequency of the signal. As a result, the minimum of the extracted frequency slope can be determined from the power spectrum, Figure 2b. It represents the position of the phase minimum which also occurs at $\lambda_{eq}$. The ROI is defined in the proximity of the detected $\lambda_{eq}$ where only unambiguous phase data is included. This so called local phase, $\varphi_{loc}$, is subject to a phase offset, Δφ, with regard to the absolute phase due to the $\cos^{-1}$-operation, Equation (3).

A second analytical step implements a newly developed approach called wrapped phase derivative evaluation (WPDE) where $\varphi_{loc}$ is differentiated with respect to the wavelength, noted with $\frac{\partial }{\partial \lambda }$,

(4) $$\varphi_{loc}^{\prime }=\frac{\partial }{\partial \lambda }\left(2\pi \frac{\left[\left(n_{smp}\left(x,\lambda \right)-1\right)t_{smp}\right]+\left[\left(n_{DE}\left(\lambda \right)-1\right)t_{DE}\right]-\delta }{\lambda }+\Delta \varphi \right).$$

Equation (4) eliminates the phase offset, Δφ, and enables the evaluation of the cross-linking characteristics in terms of the group refractive index, $n_{g}^{smp}\left(x,\lambda \right)$, or the relative derived optical thickness (RDOT) $t_{OPT}^{\prime }$

(5) $$\begin{array}{c} n_{g}^{smp}\left(x,\lambda \right)=1-\frac{\lambda^{2}\;\cdot \;\tau }{2\pi \;\cdot \;t_{smp}} \end{array}$$

(6) $$\begin{array}{c} \mathrm{with}\tau =\varphi_{loc}^{\prime }-\frac{2\pi }{\lambda^{2}}\left[\left(1-n_{g}^{DE}\right)t_{DE}+\delta \right] \end{array}$$

(7) $$\begin{array}{c} t_{OPT}^{\prime }=n_{g}^{smp}\left(x,\lambda \right)\;\cdot \;t_{smp}=t_{smp}-\frac{\lambda^{2}\tau }{2\pi } \end{array}$$

where $\varphi_{loc}^{\prime }$ is calculated from the measured data using the difference quotient with Δλ as the interval. A detailed derivation of Equations (5)–(7) can be found in the Appendix A. As the knowledge of $t_{smp}$ and $t_{DE}$ determines the accuracy of the calculation of $n_{g}$, the respective error in measurement has a significant influence on the overall error. For this purpose, a propagation of uncertainty was performed with

(8) $$\Delta n_{g}=\sqrt{{\left(\frac{\partial n_{g}\left(t_{smp}\right)}{\partial t_{smp}}\;\cdot \;\Delta t_{smp}\right)}^{2}}$$

for a case with the group refractive index of bulk materials without additional dispersion and with

(9) $$\Delta n_{g}=\sqrt{{\left(\frac{\partial n_{g}\left(t_{smp}\right)}{\partial t_{smp}}\;\cdot \;\Delta t_{smp}\right)}^{2}+{\left(\frac{\partial n_{g}\left(t_{DE}\right)}{\partial t_{DE}}\;\cdot \;\Delta t_{DE}\right)}^{2}}$$

for the determination of the group refractive index of thin materials with additional dispersion. In both cases, the uncertainty of the measurement of the thicknesses, $\Delta t_{smp}$ and $\Delta t_{DE}$ respectively, is assumed to be 4 nm for sizes smaller 1 mm and 20 nm for sizes larger 2.5 mm [34]. For the samples analyzed within this work it was calculated that the uncertainty is $\Delta n_{g}=$2.2 × 10^−6^ for bulk materials with $t_{smp}$ = 5 mm and $\Delta t_{smp}$ = 20 nm. Furthermore, the calculated uncertainty is $\Delta n_{g}=$3.4 × 10^−6^ for samples where $t_{smp}$ = 750 μm with $\Delta t_{smp}$ = 4 nm and $t_{DE}$ = 4 mm with $\Delta t_{DE}$ = 20 nm.

**Figure 3:**
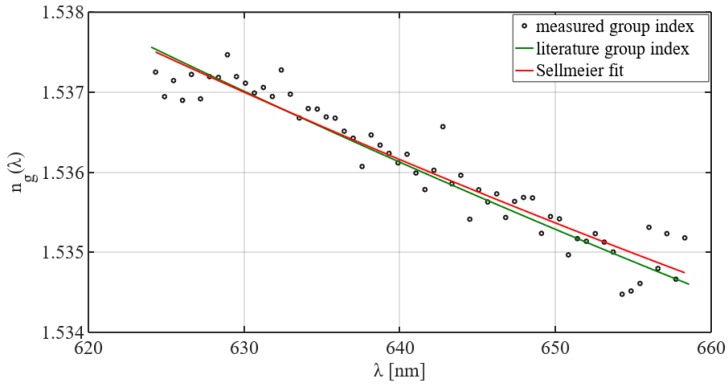
Plot of the measured group refractive index $n_{g}\left(\lambda \right)$ of N-BK7 which was calculated using the WPDE approach and its corresponding Sellmeier fit in comparison to the literature values according to [36].

In order to evaluate the algorithm, a sample of N-BK7 ($t_{smp}$ = 5 mm) was measured. The corresponding group refractive index $n_{g}\left(\lambda \right)$ was calculated and fitted using the Sellmeier equation [35], Figure 3.

In comparison to the literature values [36], a root-mean-square error for the fitted values of 7.9 × 10^−5^ is achieved. This demonstrates that the WPDE approach achieves a comparable accuracy as state-of-the-art refractive index measurement technologies. Furthermore, the refractive index resolution is sufficient to characterize cross-linking in waveguide polymers, where differences in the range of Δn= 0.001 − 0.02 are expected, taking the respective sample thickness into account [22].

As this result is calculated only within the ROI and is dependent on the amount of dispersion, represented by $n_{g}^{smp}\left(\lambda \right)$, for a small spectral range. Different approaches have been considered to gather information over the complete spectral range of the data set. On the one hand, the WPDE analysis algorithm can be applied to other ROIs within the data. The advantage is that the group refractive index can be calculated without an a priori knowledge of the underlying material model. On the other hand, one can calculate the group refractive index over the complete spectral range, if the material model of the sample is known.

## 3. Results

### 3.1. Interferometric Profile Evaluation

**Figure 4:**
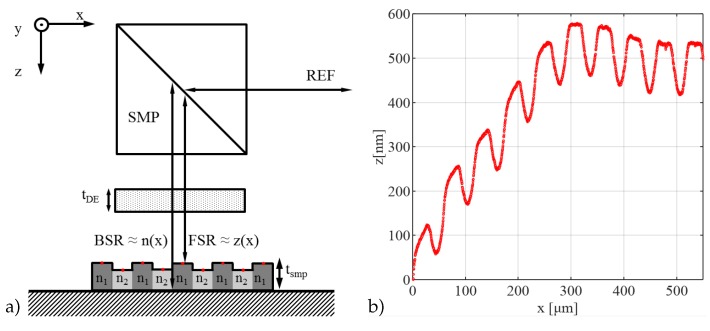
(**a**) Experimental schematic on a lithographically structured sample of the thickness $t_{smp}$ with varying refractive indices, $n_{1}$ and $n_{2}$, along the *x*-axis in the SMP—sample arm where the FSR-front surface reflex contains information on the surface height profile z(x) due to shrinkage and the BSR-back surface reflex contains information on the refractive index slope along the *x*-axis n(x) in a setup with a dispersive element of the thickness $t_{DE}$. Probe points for single RDOT measurements are marked with red dots on the sample while REF indicates the reference arm; (**b**) Plot of the measured surface profile z(x) from a polymer sample under investigation utilizing a wavelength-calibrated imaging spectrometer.

As cross-linking affects the refractive index as well as the geometrical dimensions of a sample in the form of shrinkage, a measurement setup that relies on the geometrical dimensions (e.g., thickness) has to account for shrinkage accordingly. For this purpose, the interference signal was separated according to its frequency content and the temporal occurrence of the frequency content, Figure 4a. Due to the thickness of the sample, the backreflected light from the front surface as well as from the back surface can be analysed independently. The surface height profile of the sample has been characterized with the described setup using the front-surface reflex and the method described in [30], Figure 4b.

It is obvious, that apart from a slight overall waviness, the sample shows a regular height pattern with the expected pitch of 50 μm. The depth of the shrunken areas is about 120 nm, which lies in the expected range. In consequence, these calculated height profiles enable the separation of shrinkage from the refractive index information for every sample individually and simultaneously.

### 3.2. Cross-Linking Characterization

**Figure 5:**
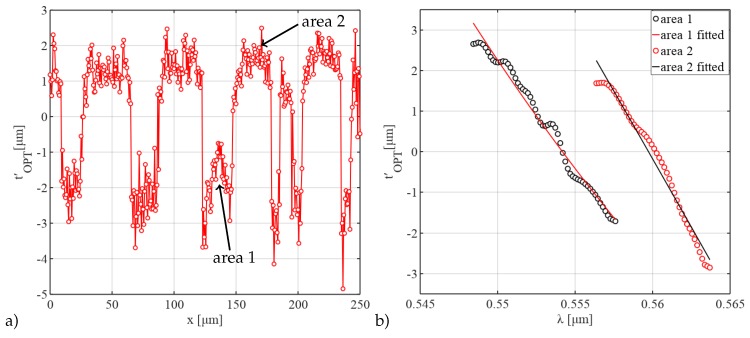
Results of the measured RDOT (**a**) spatially resolved along one sample dimension of a lithographically structured photoresist layer with structures having a nominal pitch of 50 μm on a Si-substrate at a wavelength of 557 nm and (**b**) mean values and fitted data for two marked areas with different degrees of cross-linking over a spectral range.

With the knowledge of the surface height profile of the sample due to shrinkage, the correct thickness along the spatial domain, $t_{smp}=t_{smp}\left(x\right)∼z\left(x\right)$, can be calculated. Therefore, the surface height profile was measured in reference to the substrate as shown in Figure 4a. By the application of either Equation (5) or (7), the group refractive index or the relative derived optical thickness can be calculated in relation to its position on the sample, Figure 5a.

For the results pictured above, the RDOT profile of the sample was calculated at a single wavelength of 557 nm. The spatial profile allows a resolution of cross-linking differences of 4 μm in the lateral domain. Although the results are affected by noise and batwing-effects [37], a dynamic range of ±1.5 μm in the RDOT for the given sample was revealed over a lateral range of nearly 550 μm where a section of 250 μm is displayed here. Furthermore, it was also visible that the plateaus do not show completely flat RDOT profiles. This behavior was attributed to a mixture of effects ranging from diffraction during exposure of the structures over deformation during shrinkage to diffraction during measurements. As the profile was taken at a specific wavelength, it represents only a fraction of the captured information, which was originally analyzed over a spectral range of 20 nm.

In order to estimate the effect of cross-linking, the RDOT differences as a mean value of two different exposed areas have been measured over the complete spectral range, Figure 5b. An RDOT difference of about 3 μm between the differently cross-linked areas could be resolved while the RDOT slope for every area was determined over 10 nm. The results are affected by noise in the original data which gets more pronounced by the process of taking the derivative. Some smoothing using a Gaussian filter was applied to the data.

One of the main advantages of the described approach is the lack of necessity for a model in order to calculate the spectrally-resolved refractive index. As some compromise towards the size of the spectral measurement range was being made by the choice of the dispersive element Section 2.1, the application of a refractive index model might become interesting in post-processing. In the context of (photo)polymers, a variant of Cauchy’s equation was selected [38,39]. Using this model, the group refractive index $n_{g}^{smp}\left(\lambda \right)$ can be calculated according to Delbarre et al. [40] with

(10) $$n_{g}^{smp}\left(\lambda \right)=n\left(\lambda \right)-\frac{dn(\lambda )}{d\lambda }\;\cdot \;\lambda =A+\frac{3B}{\lambda^{2}}+\frac{5C}{\lambda^{4}}.$$

By appropriate fitting, the Cauchy coefficients A,B and C have been determined which enable the calculation of the refractive index and the group refractive index over any given spectral range where the Cauchy model is valid.

## 4. Discussion and Conclusions

In order to characterize the cross-linking of waveguide polymers, an experimental approach based on a low-coherent interferometer was developed. The approach utilized the material dispersion of the sample as well as an imaging spectrometer to gather the wavelength-dependent, derived optical thickness in a spatially resolved fashion as a measure for cross-linking. To evaluate the derived optical thickness, a novel approach called WPDE was developed and successfully applied to the measurement of a negative tone resist sample. While previous works demonstrated that the fundamental method is capable of resolving differences in cross-linking degrees with high repeatability [31], the novel approach extended the method even further. It was demonstrated that the refractive index, derived from the optical thickness, can be measured with an accuracy of about 7.9 × 10^−5^ as RMS error with regard to literature values. Furthermore, optical thickness profiles over spatial dimensions of several hundred μm could be acquired in a single frame measurement requiring only 50 milliseconds acquisition time. State-of-the-art cross-linking metrology approaches are strongly limited in this regard. Additionally, the approach allows not only the measurement of refractive index/optical thickness profiles but also the determination of surface height profiles simultaneously.

The results show that the method allows for measurements of optical thickness variations in the range of ±1.5 μm and a lateral spatial resolution of about 4 μm. The WPDE analysis of one data point takes about 0.35 ms which leads to an analysis time of about 717 ms for a typical 2048 data point measurement. This provides fast, one-shot evaluation of cross-linking within the integration time of the camera. The use of this method for production accompanying tasks is favorable due to its speed. Minor problems occur measuring thin samples where signals from multiple reflections obscure the data. Algorithms to filter these signals appropriately are being worked on. Future work will be focused on the quantification of the precision, the reduction of deviations such as bat-wing effects and the acceleration of the analysis algorithms.

## Abbreviations

The following abbreviations are used in this manuscript:

DSC	Differential scanning calorimetry
FFT	Fast Fourier transform
N-BK7	Borosilicate-crown glass
RDOT	Relative derived optical thickness
ROI	Region of interest
STFT	Short-time Fourier transform
WPDE	Wrapped phase derivative evaluation

## Appendix A. Derivation of the Group Refractive Index

This sections aims to deliver additional information on the derivation of Equation (5) in the Section 2.2 based on Equation (4),

(A1) $$\varphi_{loc}^{\prime }=\frac{\partial }{\partial \lambda }\left(2\pi \frac{\left[\left(n_{smp}\left(x,\lambda \right)-1\right)t_{smp}\right]+\left[\left(n_{DE}\left(\lambda \right)-1\right)t_{DE}\right]-\delta }{\lambda }+\Delta \varphi \right).$$

For the purpose of readability, the spatially and spectrally dependent refractive index of the sample $n_{smp}\left(x,\lambda \right)$ will be noted as $n_{smp}$ and the spectrally dependent refractive index of the dispersive element $n_{DE}\left(\lambda \right)$ will be noted as $n_{DE}$ in the following equations. In the case of the first derivative, the quotient rule applies with

(A2) $$\begin{array}{c} f^{\prime }\left(x\right)=\frac{g^{\prime }\left(x\right)\;\cdot \;k\left(x\right)-g\left(x\right)\;\cdot \;k^{\prime }\left(x\right)}{k^{2}\left(x\right)} \end{array}$$

(A3) $$\begin{array}{c} g\left(x\right)=2\pi \;\cdot \;\left[\left(n_{smp}-1\right)t_{smp}+\left(n_{DE}-1\right)t_{DE}-\delta \right] \end{array}$$

(A4) $$\begin{array}{c} g^{\prime }\left(x\right)=2\pi \;\cdot \;\left[\frac{dn_{smp}}{d\lambda }\;\cdot \;t_{smp}+\frac{dn_{DE}}{d\lambda }\;\cdot \;t_{DE}\right] \end{array}$$

(A5) $$\begin{array}{c} k(x)=\lambda \end{array}$$

(A6) $$\begin{array}{c} k^{\prime }\left(x\right)=1 \end{array}$$

(A7) $$\begin{array}{c} k^{2}\left(x\right)=\lambda^{2}. \end{array}$$

This leads to

(A8) $$\begin{array}{cc} \varphi_{loc}^{\prime } & =\frac{2\pi \;\cdot \;\left[\frac{dn_{smp}}{d\lambda }\;\cdot \;t_{smp}+\frac{dn_{DE}}{d\lambda }\;\cdot \;t_{DE}\right]\;\cdot \;\lambda }{\lambda^{2}}\\ & -\frac{2\pi \;\cdot \;\left[\left(n_{smp}-1\right)t_{smp}+\left(n_{DE}-1\right)t_{DE}-\delta \right]\;\cdot \;1}{\lambda^{2}} \end{array}$$

which can be simplified and separated into two terms, *A* and *B*, for the sake of individual assessment

(A9) $$\begin{array}{cc} \varphi_{loc}^{\prime } & =\frac{2\pi \;\cdot \;\left[\frac{dn_{smp}}{d\lambda }\;\cdot \;t_{smp}+\frac{dn_{DE}}{d\lambda }\;\cdot \;t_{DE}\right]}{\lambda }\\ & -\frac{2\pi \;\cdot \;\left[\left(n_{smp}-1\right)t_{smp}+\left(n_{DE}-1\right)t_{DE}-\delta \right]}{\lambda^{2}}\\ & =A-B \end{array}$$

(A10) $$\begin{array}{c} A\equiv \frac{2\pi \;\cdot \;\left[\frac{dn_{smp}}{d\lambda }\;\cdot \;t_{smp}+\frac{dn_{DE}}{d\lambda }\;\cdot \;t_{DE}\right]}{\lambda } \end{array}$$

(A11) $$\begin{array}{c} B\equiv \frac{2\pi \;\cdot \;\left[\left(n_{smp}-1\right)t_{smp}+\left(n_{DE}-1\right)t_{DE}-\delta \right]}{\lambda^{2}} \end{array}$$

Under the assumption that the derivative of the refractive index *n*, in relation to the wavelength λ, can be described as a difference of the index and the group index $n_{g}$ following [40].

(A12) $$\frac{dn}{d\lambda }=\frac{n-n_{g}}{\lambda },$$

the term *A* can be rewritten as

(A13) $$\begin{array}{cc} A & =\frac{2\pi }{\lambda }\left[\frac{n_{smp}-n_{g}^{smp}}{\lambda }\;\cdot \;t_{smp}+\frac{n_{DE}-n_{g}^{DE}}{\lambda }\;\cdot \;t_{DE}\right]\\ & =\frac{2\pi }{\lambda^{2}}\left[\left(n_{smp}-n_{g}^{smp}\right)\;\cdot \;t_{smp}+\left(n_{DE}-n_{g}^{DE}\right)\;\cdot \;t_{DE}\right] \end{array}$$

and the term *B* can be rewritten as

(A14) $$\begin{array}{c} B=\frac{2\pi }{\lambda^{2}}\left[\left(n_{smp}-1\right)\;\cdot \;t_{smp}+\left(n_{DE}-1\right)\;\cdot \;t_{DE}-\delta \right] \end{array}$$

In consequence Equation (A9) can be expressed with

(A15) $$\begin{array}{cc} \varphi_{loc}^{\prime } & =A-B\\ & =\frac{2\pi }{\lambda^{2}}\left[\left(n_{smp}-n_{g}^{smp}\right)\;\cdot \;t_{smp}-\left(n_{smp}-1\right)\;\cdot \;t_{smp}\right.\\ & \left.+\left(n_{DE}-n_{g}^{DE}\right)\;\cdot \;t_{DE}-\left(n_{DE}-1\right)\;\cdot \;t_{DE}+\delta \right]\\ & =\frac{2\pi }{\lambda^{2}}\left[\left(n_{smp}-n_{g}^{smp}-n_{smp}+1\right)\;\cdot \;t_{smp}\right.\\ & \left.+\left(n_{DE}-n_{g}^{DE}-n_{DE}+1\right)\;\cdot \;t_{DE}+\delta \right]\\ & =\frac{2\pi }{\lambda^{2}}\left[\left(1-n_{g}^{smp}\right)\;\cdot \;t_{smp}+\left(1-n_{g}^{DE}\right)\;\cdot \;t_{DE}+\delta \right]\\ & =\frac{2\pi }{\lambda^{2}}\left(1-n_{g}^{smp}\right)\;\cdot \;t_{smp}+\frac{2\pi }{\lambda^{2}}\left[\left(1-n_{g}^{DE}\right)\;\cdot \;t_{DE}+\delta \right]\\ \varphi_{loc}^{\prime }-\frac{2\pi }{\lambda^{2}}\left[\left(1-n_{g}^{DE}\right)\;\cdot \;t_{DE}+\delta \right] & =\frac{2\pi }{\lambda^{2}}\left(1-n_{g}^{smp}\right)\;\cdot \;t_{smp}. \end{array}$$

For readability the left part of the equation is substituted with τ and solved for the group refractive index of the sample, $n_{g}^{smp}$, which equals Equations (5) and (7) of the main text of the paper, whereas it has to be noted that $n_{smp}=n_{smp}\left(x,\lambda \right)$ and $n_{DE}=n_{DE}\left(\lambda \right)$:

(A16) $$\begin{array}{c} \tau =\varphi_{loc}^{\prime }-\frac{2\pi }{\lambda^{2}}\left[\left(1-n_{g}^{DE}\right)t_{DE}+\delta \right] \end{array}$$

(A17) $$\begin{array}{c} \mathrm{and}\;n_{g}^{smp}=1-\frac{\lambda^{2}\;\cdot \;\tau }{2\pi \;\cdot \;t_{smp}}. \end{array}$$
